# Paradoxical consequences of wastewater interventions targeting carbapenemase-producing Enterobacterales

**DOI:** 10.1017/ash.2023.393

**Published:** 2023-09-29

**Authors:** David Lehman, Shireen Kotay, Hardik Parikh, Stacy Park, Amy Mathers

## Abstract

**Background:** *Serratia marcescens* is a leading cause of hospital-acquired infections. There has been increasing recognition of hospital wastewater as a reservoir for carbapenemase-producing Enterobacterales (CPE), including *S. marcescens*. Because CPE can proliferate in biofilms in sink drains and traps, controlling nosocomial spread is challenging. The ideal approach to eliminate transmission from wastewater to patients remains unknown. **Methods:** Patients were included if they were admitted to 1 of 2 intensive care units (ICUs) for >12 hours between December 1, 2010, and January 31, 2016. During this period at the University of Virginia Hospital, there was ongoing patient acquisition of multiple species producing *Klebsiella pneumoniae* carbapenemase (KPC) as well as consistent perirectal KPC surveillance. In January 2014, to eliminate CPE-colonized sinks, the sink drains and traps in one of the ICUs (ie, the “intervention unit”) were exchanged followed by varied chemical mitigations to prevent recolonization. In another ICU, the same chemical mitigations were performed but without plumbing replacement (ie, the “control unit”). Acquisition of KPC-producing *S. marcescens* was defined as colonization or infection >12 hours after admission to either unit. To control for increases in patient-to-patient transmission, acquisition of methicillin-resistant *Staphylococcus aureus* (MRSA) was evaluated in the intervention unit during the same period and was defined as new colonization or infection with MRSA >12 hours after unit admission but within 21 days of last unit exposure. **Results:** For the postintervention period, risk of *S. marcescens* acquisition was increased (RR, 2.85; 95% CI, 1.24–6.58; *P* = .01) in the intervention unit compared to the control unit. In the intervention unit, the risk of *S. marcescens* acquisition increased in the postintervention period compared to the preintervention period (RR, 6.26; 95% CI, 2.59–15.1; *P* < .0001). There was no change in MRSA acquisition in the intervention unit representing consistent patient-to-patient infection prevention (RR, 0.95; 95% CI, 0.61–1.48; *P* = .81). *S. marcescens* isolates were noted to be highly clonal. **Conclusions:** Exposure to the intervention unit following plumbing replacement was associated with increased relative risk of acquisition of KPC-producing *S. marcescens*. This increased risk was not observed in the control unit, which had only chemical plumbing interventions. There was no concomitant increase in patient-to-patient MRSA transmission. The disturbance of the wastewater environment through the plumbing replacement intervention may have led to the unintended consequence of more KPC-producing *S. marcescens* acquisition.

**Disclosures:** None

